# Efficacy and Tolerability of Methotrexate for Idiopathic Granulomatous Mastitis: A Systematic Review and Meta‐Analysis

**DOI:** 10.1155/tbj/6710172

**Published:** 2026-02-13

**Authors:** Ying Han, LiHui Shi, YanRan Zhang

**Affiliations:** ^1^ Department of Science and Education, Tongzhou Maternal and Child Health Hospital of Beijing, Beijing, 101100, China; ^2^ Department of Breast Surgery, Tongzhou Maternal and Child Health Hospital of Beijing, Beijing, 101100, China

**Keywords:** complete remission, idiopathic granulomatous mastitis, meta-analysis, methotrexate, recurrence, side effects

## Abstract

**Background:**

Idiopathic granulomatous mastitis (IGM) is a challenging inflammatory breast disease with limited standardized treatment guidelines.

**Methods:**

This meta‐analysis systematically evaluated the efficacy and safety of methotrexate (MTX) in IGM, pooling data from observational studies. We assessed complete remission rates, recurrence rates, and severe side effects leading to MTX discontinuation using both common and random effects models, accounting for heterogeneity.

**Results:**

Nine studies were included. In analyses without control groups, the pooled complete response rate for MTX was 61.6% (95% CI: 40.4–79.2%), with significant heterogeneity (*I* ^2^ = 81.4%). Combination therapy showed a higher complete response rate in the common effect model (78.0%, 95% CI: 71.4–83.3%) compared to monotherapy (46.6%, 95% CI: 34.2–59.3%), though this difference was not significant in the random effects model. The overall pooled proportion of severe side effects leading to MTX discontinuation was low at 1.23% (95% CI: 0.40–3.74%) in the common effect model and 0.51% (95% CI: 0.03–9.37%) in the random effects model, with no significant heterogeneity. In controlled studies, MTX showed no significant difference in complete remission compared to steroid‐containing controls.

**Conclusion:**

Based on a pooled complete response rate of 61.6%, MTX appears to be an effective and well‐tolerated treatment for IGM, though substantial heterogeneity exists in uncontrolled studies.

## 1. Introduction

Granulomatous mastitis (GM), a broad term encompassing several inflammatory conditions of the breast, is a rare, chronic inflammatory breast condition primarily affecting women of childbearing age. The term encompasses both specific etiologies (e.g., infectious, autoimmune, or foreign‐body‐related) and idiopathic forms. Idiopathic granulomatous mastitis (IGM) is a subtype of GM where other known etiologies, such as infectious or systemic diseases, have been ruled out [1, 2]. IGM, by definition, is a diagnosis of exclusion applied only after ruling out identifiable causes such as tuberculosis, sarcoidosis, fungal infections, ductal ectasia, or malignancy [3–6]. IGM predominantly affects women of reproductive age (typically 20–40 years), with a reported association with recent pregnancy, lactation, hyperprolactinemia, or oral contraceptive use, though the exact pathogenesis remains. The condition mimics breast cancer or infectious mastitis, complicating timely diagnosis [7]. Clinically and radiologically, IGM mimics inflammatory breast carcinoma, leading to diagnostic delays and unnecessary mastectomies in some cases.

Importantly, GM and IGM are not interchangeable entities. GM is a broader pathological descriptor that includes IGM as its idiopathic subset. Throughout this manuscript, we use “GM” only when referring to the general category and “IGM” exclusively for the idiopathic form under study, in line with expert consensus [3–6]. Akbulut et al. have repeatedly emphasized this distinction, noting that conflating the terms leads to flawed epidemiological analyses and treatment algorithms [5, 6]. For instance, infectious GM (e.g., corynebacterial) responds to antibiotics, whereas IGM requires immunosuppression; misclassification inflates reported “recurrence” rates in pooled GM series [3].

No standardized treatment exists for IGM owing to its rarity and etiological uncertainty. Options range from observation and aspiration to wide surgical excision, but recurrence after surgery exceeds 50% in some series [8]. Based on existing literature, several management strategies are proposed, including corticosteroids, immunosuppressive therapies, and surgical remedies; nonetheless, no standard protocol exists due to the rare nature of the disease and heterogeneity in study designs [8, 9]. Corticosteroids remain first line but carry risks of dependency, relapse upon tapering, and metabolic sequelae [10]. MTX is an immunosuppressive drug that has shown potential for the treatment of IGM and is used alone or in combination with corticosteroids to reduce inflammation as well as promote remission [8, 10]. A folate antagonist with proven efficacy in steroid‐refractory autoimmune granulomatous diseases has gained traction as a steroid‐sparing agent in IGM [3, 4]. Retrospective data suggest that MTX promotes remission by inhibiting T‐cell proliferation, reducing cytokine release, and attenuating granuloma formation, with acceptable toxicity at low weekly doses.

Though MTX is being increasingly used, previous studies on it for IGM have many limitations. Most of them were small, retrospective, or single‐arm studies, often without control groups or standardized outcome measures, undermining robust conclusions on efficacy, recurrence rates, and safety [1, 7, 9, 11–13]. Most reports are small (*n* < 50), single‐arm, and retrospective, with inconsistent definitions of remission (clinical vs. radiographic) and recurrence (during vs. post‐treatment). Monotherapy versus combination with steroids further clouds comparative efficacy. To illustrate, the distinguished experts say that there are reports suggesting that MTX may decrease the chances of recurrence while helping achieve complete remission, but there are others that refer to variable response rates and different adverse effect profiles, with very little delineation on the effect of using monotherapy as opposed to combination therapy [11, 14]. Akbulut’s systematic reviews of > 3,000 GM cases highlight that < 5% received MTX, precluding robust subgroup analysis [5]. The absence of comprehensive meta‐analyses synthesizing global data further limits evidence‐based decision‐making. Additionally, studies often fail to account for variations in MTX dosing, administration routes (oral vs. subcutaneous), and follow‐up durations, leading to conflicting findings on optimal treatment strategies [2, 8]. No prior meta‐analysis has synthesized MTX‐specific outcomes in histologically confirmed IGM while addressing these methodological gaps. The existing gaps call for systematic assessment to elucidate the role of MTX in the management of IGM.

This meta‐analysis addresses these deficiencies by synthesizing data from diverse, international observational studies exclusively in histologically confirmed IGM (excluding known‐etiology GM) to evaluate MTX’s efficacy in complete remission and recurrence, alongside safety in terms of severe adverse events necessitating discontinuation. Through monotherapy versus combination therapy subgroup analyses, we directly respond to Akbulut et al.’s emphasis on precise conceptual separation of IGM from broader GM and the need for robust MTX‐specific evidence, aiming to provide a clearer evidence base for clinical practice and future standardized protocols.

## 2. Materials and Methods

The meta‐analysis and systematic review were performed according to the PRISMA guidelines [11]. The study was preregistered in the PROSPERO international prospective register of systematic reviews (registration number: CRD420251156715).

### 2.1. Literature Search

From as far back as inception, the systematic search for the literature was carried out across the PubMed, Embase, and Web of Science databases until April 8, 2025. The search strategy was created to find studies on the safety and efficacy of MTX in treating IGM. It combined medical subject heading (MeSH) terms, Emtree terms, and free‐text keywords regarding the condition, intervention, and outcomes. Specific search strings were developed for each database. The PubMed search string was (“Mastitis, Granulomatous”[MeSH] OR “idiopathic granulomatous mastitis” OR “granulomatous lobular mastitis”) AND (“Methotrexate”[MeSH] OR “Methotrexate” OR “MTX”). The Embase search string was (“idiopathic granulomatous mastitis”/exp OR “idiopathic granulomatous mastitis” OR “granulomatous mastitis”) AND (“methotrexate”/exp OR “methotrexate” OR “mtx”). The Web of Science search string was ALL = ((“granulomatous mastitis” OR “idiopathic granulomatous mastitis” OR “granulomatous lobular mastitis”) AND (“methotrexate” OR “MTX”)). The search was also performed in the Cochrane Database of Systematic Reviews and Scopus using the following adapted search strategies: Cochrane: (“granulomatous mastitis” OR IGM) AND (methotrexate OR MTX) in Title, Abstract, or Keywords. Scopus: TITLE‐ABS‐KEY((“granulomatous mastitis” OR IGM) AND (methotrexate OR MTX)).

### 2.2. Study Selection

This review defined its eligibility criteria using the Participant, Intervention, Comparator, Outcomes, and Study design (PICOS) framework, systematically screening and selecting studies based on histopathologically confirmed IGM patients treated with MTX and reporting relevant outcomes from eligible observational studies. Studies were included if they met pre‐established eligibility criteria. Duplicate records were identified and eliminated using reference management software. To select a set of articles, two steps were involved: screening of titles and abstract and then the full‐text screening.

• Patient: Inclusion criteria were that the study needed to concern patients with histopathologically proven IGM and no other granulomatous disease or undiagnosed cases.
• Intervention: The inclusion criteria were that the study had to assess the treatment of IGM with MTX. The studies were weeded out in case they looked at other interventions or MTX was not the focus of treatment.
• Outcome to measure: Only studies involving outcomes in terms of recurrence, complete remission, or severe side effects that resulted in discontinuation of MTX were taken. The research papers were filtered out on the basis of not having these results or not having enough data to do so.
• Design of studies: The studies were considered in case they were observational studies, e.g., retrospective cohort studies. The inclusion criteria were that studies had to be original research studies and not reviews, or editorials, or any other type of nonoriginal research.

Titles and abstract screening were done by two independent reviewers according to inclusion and exclusion criteria after which full‐text articles were screened. A discrepancy was solved by discussing or consulting with a third reviewer.

### 2.3. Data Extraction

Data were extracted using a pilot tested and standardized data extraction form by two independent reviewers. The data obtained consisted of the characteristics of the studies (first author, year of publication, country, study design, and the sample size) as well as patient demographics (age range and diagnostic criteria), intervention (dose of MTX, route of administration, duration of treatment, and combination therapy), and outcomes (complete remission, recurrence, severe adverse events, number of events, and follow‐up). The disagreed data extractions were addressed either by discussion or by consulting a third reviewer.

### 2.4. Quality Assessment

The quality of methodology of the included observational studies was evaluated by the use of the Newcastle–Ottawa Scale (NOS). They include the areas of selecting study participants, the comparability of groups, and evaluation of results as measured in this scale to further determine the strength of our systematic review methodology. We also followed the Assessing the Methodological Quality of Systematic Reviews (AMSTAR) guidelines. Some of the important AMSTAR items that have been used are a priori design registration, extensive literature search, identifying and extracting duplicate studies and data, and risk of bias assessment of studies included.

### 2.5. Statistical Analysis

Statistical tests were conducted with R software (Version: 3.2.1) [12]. The meta‐analysis model was used to compute the pooled proportions or odds ratios of the primary outcomes (recurrence, complete remission, and severe side effects). The decision to use common and random effects models was made after evaluating the heterogeneity with *I* ^2^ statistic. Sensitivity analyses have been used to examine how the pooled estimates were affected by particular characteristics of the studies.

The *I* ^2^ statistic and Cochran Q test were used to determine statistical heterogeneity. The assessment of publication bias was conducted with the help of funnel plots and Eggers test. Subgroup analyses were conducted to explore possible causes of heterogeneity and to test the impacts of particular study attributes, e.g., combination therapy compared to monotherapy. Where applicable, linear regression models were employed to investigate the association between the study characteristics and outcomes. Any statistical analysis was done at a significance level of *p* < 0.05.

## 3. Results

### 3.1. Meta‐Analysis Study Selection

**FIGURE 1:**
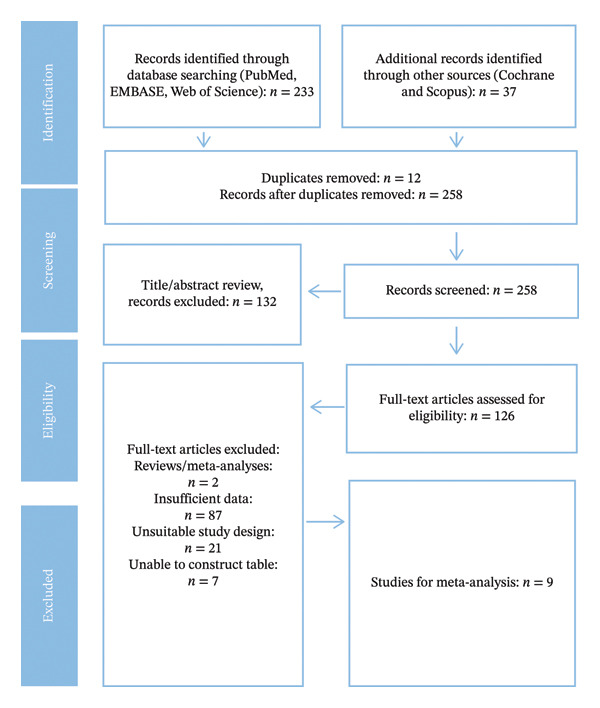
Characteristics of the included studies.

The selection of the study was conducted in accordance with PRISMA, starting with 233 records found by search in PubMed, EMBASE, and Web of Science. Another 37 records were found in Cochrane and Scopus. Upon eliminating 12 duplicates, 258 unique records were filtered by title and abstract, resulting in 132 being discarded. The rest of the 126 full‐text articles were screened on the basis of eligibility. One hundred and seventy studies were excluded based on the reasons such as reviews (*n* = 2), lack of enough data (*n* = 94), and inappropriate study design (*n* = 21). The final outcome, the meta‐analysis encompassed nine studies that passed the inclusion criteria and were incorporated in the analysis (Figure 1).

The studies that are included have a wide spectrum of features, with most of them being conducted in the form of retrospective cohort designs to establish the effectiveness of MTX in the treatment of IGM. The geographical coverage of the studies includes Turkey, Iran, Iraq, China, and the United States, which demonstrates the worldwide interest in this condition. The size of samples used is quite diverse, between 10 and 81 patients; the regimen of MTX usage varies between 5 mg per week to 25 mg per week with the addition of corticosteroids given orally or subcutaneously. Follow‐up periods also differ, spanning from a minimum of 2 months to several years, with varying definitions of remission and relapse. Patient age ranges reflect women of childbearing age, generally between 18 and 65 years, though some studies provide only mean ages or specific ranges (Table 1). The methodological quality of this meta‐analysis, as assessed by the AMSTAR 2 checklist, was rated as moderate, with no critical flaws (Table 2).

**TABLE 1:** Characteristics of the studies meeting the inclusion criteria.

Study	Country	*n*	MTX protocol	Follow‐up	Age range
Nawzad Khaleil et al. [13]	Iraq	13	7.5 mg daily/QOD for 6 mo; combined w/low‐dose steroids.	≥ 2 mo after Tx	N/A
Fereshte et al. [14]	Iran	12	7.5–10 mg/wk, up to 15 mg/wk; alone or w/prednisone 10–15 mg/d.	Mean 11.9 ± 4.4 mo (6–22 mo); clinical F/U Q1‐3 wks.	23–47 yrs
Ahmad et al. [15]	Iran	10	N/A	N/A	34.06 ± 6.7 yrs
Demet Yalcin et al. [16]	Turkey	33	7.5–15 mg/wk; SC switch if no response/intolerance.	Tx planned for 24 mo; F/U Q2‐4 wks. Relapse defined as recurrence ≥ 3 mo post‐Tx.	18–65 yrs
Erkan et al. [17]	Turkey	62	15 mg/wk PO; SC switch/dose increase to 25 mg/wk if no response/intolerance.	F/U Q3wks; 24 mo post‐remission Tx.	28–54 yrs
Kundaktepe Berrin et al. [18]	Turkey	64	7.5–25 mg/wk; median 15 mg/wk PO; SC switch if intolerant.	Median 780d (831 ± 547d); ≥ 6 mo Tx.	23–49 yrs
Postolova et al. [19]	USA	19	10–15 mg/wk up to 20–25 mg/wk PO/SC.	Median Tx 13–15 mo (1–30 mo). F/U median 3 yrs (1–7 yrs).	Mean 33.5 yrs
Mehmet Tolga et al. [20]	Turkey	17	5 mg/wk added if partial/no response to steroids (32 mg/d prednisone).	Response criteria: 3 wk maintenance of Sx relief, normal labs, mass shrinkage.	N/A
Chunxiang et al. [21]	China	81	7.5 mg/wk for steroid‐resistant pts; w/low‐dose methylprednisolone (4–8 mg/d).	≥ 3 mo F/U.	N/A

**TABLE 2:** Critical appraisal of the meta‐analysis based on AMSTAR 2.

Standard	Assessment	Rationale
Q1: PICO components?	Yes	The review question is clearly defined with all PICO elements: Population (patients with IGM), Intervention (MTX), Comparator (implicit, other treatments or no treatment), and Outcome (recurrence, remission, side effects).
Q2: Protocol registered?	Yes	The report explicitly states the study was preregistered in a publicly accessible registry. This is a crucial step that prevents reporting bias and meets a critical standard.
Q3: Explanation of study designs?	Yes	The report provides a clear rationale for including observational study designs (retrospective cohorts, etc.) and justifies the exclusion of others (e.g., reviews).
Q4: Comprehensive search?	Yes	The search strategy is comprehensive, including multiple major databases (PubMed, EMBASE, Web of Science, Cochrane, Scopus), keyword and controlled vocabulary terms, and additional sources like reference lists and gray literature.
Q5: Duplicate study selection?	Yes	The study selection process was performed in duplicate by two independent reviewers, with a third reviewer resolving discrepancies.
Q6: Duplicate data extraction?	Yes	Data extraction from the included studies was performed independently by two reviewers using a standardized form.
Q7: List of excluded studies?	Yes	A full‐text exclusion list is provided, with reasons for exclusion (e.g., “insufficient data,” “unsuitable study design”).
Q8: Adequate detail of included studies?	Yes	The included studies are described in adequate detail, including populations, interventions (dosing, route), comparators, outcomes, and follow‐up periods.
Q9: RoB assessment?	Yes	The methodological quality of included studies was assessed using a satisfactory tool, the Newcastle–Ottawa Scale (NOS). This addresses a critical flaw and demonstrates a rigorous approach to assessing potential bias.
Q10: Funding sources?	Yes	The report explicitly mentions that the sources of funding for the included studies were reported. This addresses a critical flaw.
Q11: Appropriate statistical methods?	Yes	Appropriate statistical methods for meta‐analysis were used (random effects model, subgroup analysis) and justified, including an investigation of heterogeneity.

**Standard**	**Assessment**	**Rationale**

Q12: Impact of RoB on results?	Yes	The study’s discussion addresses the potential impact of the methodological limitations (e.g., heterogeneity, single‐arm studies) on the results, which is a key requirement for a “Yes” rating. This addresses a critical flaw.
Q13: RoB in interpretation?	Yes	The discussion provides a satisfactory explanation and discussion of heterogeneity, exploring its sources in detail (e.g., varying protocols, definitions) and acknowledging its impact on the results.
Q14: Heterogeneity explained?	Yes	The discussion provides a satisfactory explanation and discussion of heterogeneity, exploring its sources in detail (e.g., varying protocols, definitions) and acknowledging its impact on the results.
Q15: Publication bias?	Yes	The study evaluated publication bias using graphical methods (funnel plots) and statistical tests (Egger’s test), with the results discussed in the paper.
Q16: Conflicts of interest?	Yes	The report transparently discloses any potential conflicts of interest, including funding for the review itself.

### 3.2. Meta‐Analysis of MTX Treatment and Recurrence in IGM

It was a meta‐analysis that involved four datasets that were aimed at replicating the recurrence rate of IGM treated with MTX. In general, the results showed that there was a statistically significant correlation between MTX treatment and the high risk of recurrence with a pooled odds ratio (OR) of 3.15 (95% CI: 1.38–7.22) in the common effect model and 3.22 (95% CI: 1.32–7.86) in the random effects model (*p* < 0.05). The studies were not heterogeneous (*I* ^2^ = 0.0%).

**FIGURE 2:**
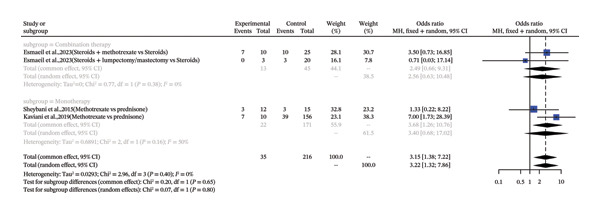
Forest plot of subgroup analysis on MTX therapy (combination vs. monotherapy) and recurrence of IGM.

The subgroup analysis according to the use of MTX as the combination or monotherapy showed the following: the combination therapy subgroup (two datasets) displayed an OR of 2.49 (95% CI: 0.66–9.31), whereas the monotherapy subgroup (two studies) had an OR of 3.68 (95% CI: 1.26–10.76). The subgroup differentiation test did not indicate any statistically significant difference between the two groups (*p* = 0.65). Equally, in the random effects model, the combination therapy subgroup revealed an OR of 2.56 (95% CI 0.63–10.48), and the monotherapy subgroup reported an OR of 3.40 (95% CI 0.68–17.02) and no significant difference was observed between subgroups (*p* = 0.80) (Figure 2).

**FIGURE 3:**
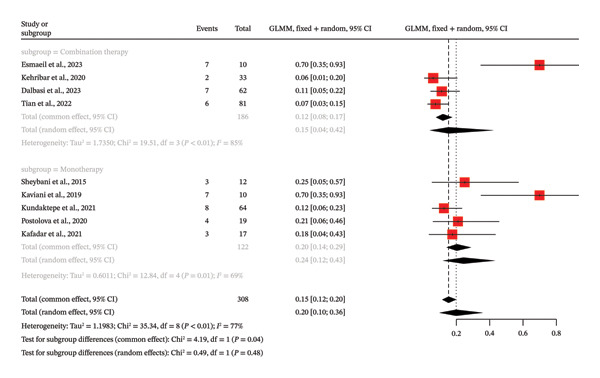
Forest plot of subgroup analysis on MTX therapy (combination vs. monotherapy) and recurrence of IGM without control groups.

The meta‐analysis method reviewed the frequency of the recurrence in patients receiving IGM treated with MTX, nine of which were not controlled, and the percentage of patients who have a recurrence was the focus of the research. The general analysis showed the high degree of heterogeneity among the researches (*I* ^2^ = 77.4%). The proportion of recurrence was estimated to be 0.1989 (95% CI: 0.0997–0.3576), which showed that about 19.9% of patients who were treated with MTX experienced recurrence, using a random effects model. Subgroup analysis was done depending on the use of MTX as combination therapy or monotherapy. According to the common effect model, the combination therapy subgroup (four studies) exhibited a recurrence proportion of 0.1183 (95% CI: 0.0792–0.1731), whereas the monotherapy subgroup (5 studies) had a recurrence proportion of 0.2049 (95% CI: 0.1424–0.2857). Subgroup difference test in the common effect model gave a *p* value of 0.0408, which implied that the difference between the two groups is statistically significant. Nevertheless, the subgroup differences were not statistically significant (*p* = 0.4826) when the random effects model was used that can explain the high heterogeneity. The proportion of recurrence was 0.1518 (95% CI: 0.0422–0.4209) and 0.2436 (95% CI: 0.1211–0.4294) in combination therapy and monotherapy, respectively, as per random effects model (Figure 3). The Egger test of the funnel plot asymmetry produced a *p* value of 0.0862, which is not significant.

### 3.3. Meta‐Analysis of MTX Treatment and Complete Remission in IGM

**FIGURE 4:**
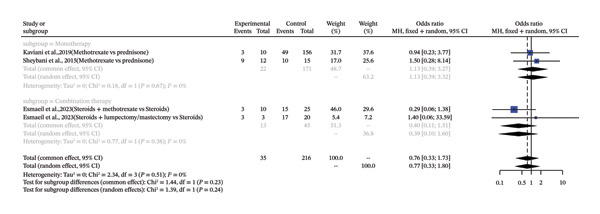
Forest plot of subgroup analysis on MTX therapy (combination vs. monotherapy) and complete remission of IGM.

This meta‐analysis examined the frequency of IGM treated with MTX, and it involved nine single‐arm studies to discuss the percentage of patients who recur. The analysis revealed that there was a high level of heterogeneity among the studies (*I* ^2^ = 77.4%). Using a random effects model, pooled recurrence proportion was estimated to be 0.1989 (95% CI: 0.0997–0.3576), which implies that about 19.9 proportion of patients treated with MTX recurred. The stratified subgroup analysis, according to the type of treatment (monotherapy vs. combination therapy) was not able to prove any significant differences also. The nonsignificant OR of 1.13 (95% CI: 0.39–3.27) and nonsignificant OR of 0.40 (95% CI: 0.11–1.51) were found in the monotherapy subgroup (*k* = 2) and the combination therapy subgroup, respectively. The subgroup difference test was not found to be statistically significant (*p* = 0.23), which proves that the selection of either monotherapy or combination therapy did not affect the outcome (Figure 4).

**FIGURE 5:**
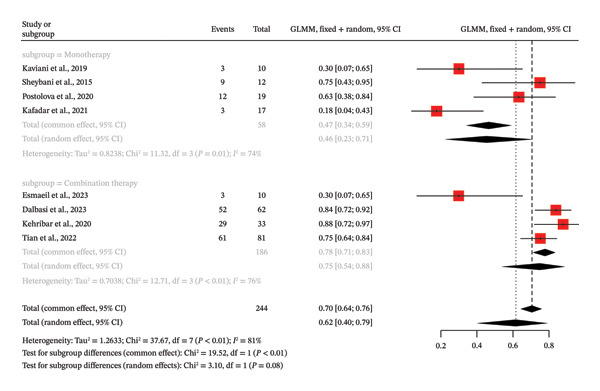
Forest plot of subgroup analysis on MTX therapy (combination vs. monotherapy) and complete remission of IGM without control groups.

This meta‐analysis relied on the evidence of eight single‐arm studies (*k* = 8) in the assessment of the entire response rate of MTX in the treatment of GM. The overall complete response rate was estimated at 61.6% based on a random effects model (95% CI: 40.4%−79.2%). Nevertheless, this general finding is characterized by a very high degree of heterogeneity, which is shown by a high *I* ^2^ value of 81.4% (*p* = 0.0001). Subgroup analysis comparing monotherapy and combination therapy showed that there was a significant difference in complete response rates under the common effect model (*p* < 0.0001). Complete response rate of combination therapy subgroup (*k* = 4) was higher than that of monotherapy subgroup (*k* = 4) with a rate of 78.0% (95% CI: 71.4%–83.3%) and 46.6% (95% CI: 34.2%–59.3%), respectively. Although this difference was present in the random effects model (combination therapy: 75.0, monotherapy: 45.7), the *p* value of the test of subgroup differences was not significantly significant (*p* = 0.0782), probably because there was excessive heterogeneity in each subgroup (*I* ^2^ > 70%) (Figure 5). The *p* value of the funnel plot asymmetry test conducted by Egger was 0.1108, which is not significant.

### 3.4. Meta‐Analysis of MTX Treatment and Side Effects in IGM

**FIGURE 6:**
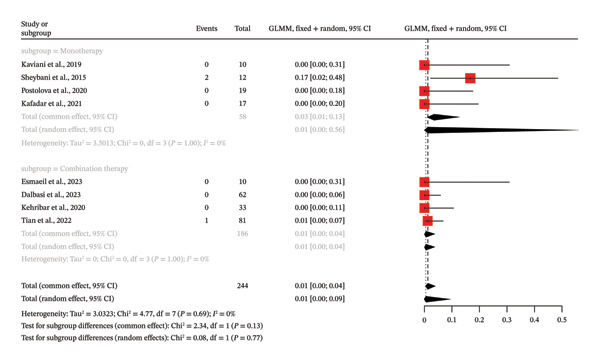
Forest plot of subgroup analysis on MTX therapy (combination vs. monotherapy) and side effects of IGM without control groups.

This is a meta‐analysis that included eight single‐arm studies (*k* = 8) with a total of 244 observations and assessed the proportion of patients with severe adverse drug reaction (ADRs) of MTX treatment to IGM. The proportion of severe ADRs in the overall pool was found to be extremely low; it was estimated to be 0.5% in the random effects model (95% CI: 0.03%–9.37%). One of the most important results is that there is no substantial heterogeneity between the studies, as has been shown by the low *I* ^2^ of 0.0% and nonsignificant *Q*‐test (*p* = 0.6883), showing that the findings of the various studies are very similar. The stratified subgroup analysis of monotherapy and combination therapy did not yield statistically significant difference between the groups as well (*p* = 0.1261 common effect model; *p* = 0.7715 random effects model). In particular, the proportion of severe ADR was 3.45 in the monotherapy group (*k* = 4) and even lower in the combination therapy group (*k* = 4); however, the difference was not significant. The unstructured event data are a true testament to the infrequency of severe ADRs as only 3 of the 244 patients had something to report and a number of the studies reported zero events. The consistency in the findings of the various models and the low heterogeneity would indicate that MTX is a safe therapy to use in the management of this condition whether as monotherapy or in combination with other medications (Figure 6). An asymmetry of funnel plot test produced a *p* value of 0.0828, and this does not indicate statistical significance, meaning there is no indication of publication bias.

## 4. Discussion

A major challenge in this meta‐analysis is the significant heterogeneity observed in the pooled proportion of recurrence, with an *I* ^2^ value of 77.4%. This high heterogeneity suggests that the included studies are not measuring the same underlying effect, and their results cannot be reliably combined. A primary reason for this heterogeneity is the vast difference in treatment protocols and patient populations across the studies. Specifically, the studies categorized under “combination therapy” and “monotherapy” are not uniform in their approach. In the combination therapy subgroup, one study [20] used a fixed‐dose combination of MTX 15 mg/week with methylprednisolone 8 mg/day, increasing doses in unresponsive patients. In contrast, another study [13] used MTX 7.5 mg/week combined with a lower dose of methylprednisolone (4–8 mg/day) but only for patients with corticosteroid‐resistant IGM. This preselected, treatment‐resistant population likely has a different disease profile and a higher inherent risk of recurrence, which could inflate their recurrence rates and introduce significant variability. Similarly, in the monotherapy subgroup, one study [14] treated patients with MTX alone, starting at 10–15 mg/week and escalating to 20–25 mg/week, for a median of 13–15 months. The other study [14] used MTX as a steroid‐sparing agent or for patients with recurrence during steroid tapering, with an MTX dose of 7.5–10 mg/week. The distinct patient selection (recurrent cases), treatment goals (steroid‐sparing), and dosing strategies likely contribute to the observed high heterogeneity. In addition, the heterogeneity is resulted by the absence of standard definitions of key outcomes and different periods of follow‐up. As an example, recurrence is characterized differently in studies. There are those who define it as a relapse during treatment and there are those who define it as post‐treatment relapse. The median follow‐up durations differ widely, with one study [14] containing a median of 11.9 months, another study [13] has its median of 18 months, and the mean of 24 months [20] with a study [15] having an amazing average of 3 years. The increased time of the follow‐up provides increased chances of recurrence, contributing to the heterogeneity.

Interestingly, the meta‐analysis that compared MTX treatment to a control group did not find any significant heterogeneity (*I* ^2^ = 0.0%). It can be probably attributed to the limited number of studies used in that analysis (only four datasets) and the fact that the control groups might have offered a more stable baseline on which comparison was performed. Nonetheless, the subgroup analysis of the two datasets according to the option of monotherapy versus the combination therapy only still had two datasets each, and the high heterogeneity of the meta‐analysis proportion of recurrence shows that even in these small subgroups the underlying patient characteristics, disease severity, treatment regimens, and follow‐up procedures are radically different and predispose to give different results that are hard to synthesize.

The observed high heterogeneity of the meta‐analysis of complete response rates to MTX in IGM in eight single‐arm studies (*I* ^2^ = 81.4, *p* < 0.0001) can be explained by a number of interrelated factors that are the result of methodological, clinical, and demographic differences between the eight included studies, which all lead to inconsistent reporting of the outcomes and responses to the treatment as reflected in the pooled complete response rate of 61.6% (95% CI: 40.4–79.2). It is also important to note that patient selection criteria differed significantly with some studies only including corticosteroid‐resistant cases such as Tian et al. where 81 patients were offered MTX at 7.5 mg/week alongside low‐dose methylprednisolone (4–8 mg/day) after the initial corticosteroid therapy and in Postolova et al. where 19 nonresistant patients were given MTX at 10–15 mg/week. There was a large degree of heterogeneity in MTX protocols. The studies varied in applying doses ranging between 5 mg/week and 25 mg/week and routes of administration. The variations added to the inconsistent folic acid supplementation and adjunctive agents may affect bioavailability and outcomes. An example is the 84% complete remission with combination therapy in Dalbasi et al. to a lower dose in Kafadar et al. to 18%. This is further supported by subgroup analyses where combination therapy subgroups (*k* = 4) have a higher complete response rate of 78.0% (95% CI: 71.4%–83.3%) in the common effect model than monotherapy in 46.6% (95% CI: 34.2%–59.3%), although with high within‐subgroup heterogeneity (*I* ^2^ > 70), suggesting that despite efforts to make it an objective criterion, even after stratification, factors such as inconsistent definitions of “complete response” across studies remained a significant contributor to heterogeneity. Also, variability was increased by differential follow‐up periods, with the shortest durations of 3 months in Tian et al. (median 18 months) and Kafadar et al. and longest of 24 months planned in Kehribar et al. and 3 years median in Postolova et al. because relapse was variably defined (e.g., symptoms > months post‐treatment in Kehribar et al.). Heterogeneity was also likely due to geographic and demographic factors since Turkey (e.g., Dalbasi et al.; Kehribar et al.; Kafadar et al.; Kundaktepe et al.), although not the only country, studied heterogeneous populations aged 18–65 years, and high response rates (84–88%) were more likely to be observed in these countries, perhaps because of comparable ethnic predispositions or healthcare behavior. This was further exacerbated by sample size differences, with smaller cohorts (such as Kaviani et al., *n* = 10) and Esmaeil et al., 2023 (*n* = 13) more likely to be biased and have larger confidence intervals than larger ones such as Tian et al. (*n* = 81), which may be skewed by a small number of studies. The study designs also played a part since most of them were of retrospective nature (e.g., Postolova et al.; Dalbasi et al.; Tian et al.) and their retrospective nature created the issue of recall bias and incomplete information compared to the prospective design used by Sheybani et al. (2015), which may lead to more consistent monitoring but still heterogeneous results. In contrast, the companion meta‐analysis that evaluated the effect of MTX on complete remission with odds ratios of four studies showed minimal heterogeneity (*I* ^2^ = 0.0%, *Q*‐test *p* = 0.5054), most likely due to the more standardized designs of the comparative studies (a total of 251 patients, 109 events), which used more standardized design, some of which used matched or randomized designs and resulted in a homogeneous pooled odds ratio of 0.76 (95% CI: 0.33–1.80, *p* = 0.54) and nonsignificant subgroup differences (*p* = 0.23) between monotherapy (OR = 1.13, *k* = 2) and combination (OR = 0.40, *k* = 2); this uniformity may stem from fewer included studies reducing variance, more rigorous inclusion criteria ensuring similar patient baselines (e.g., nonresistant cases), consistent outcome definitions aligned with control comparisons, and potentially overlapping geographic or demographic profiles, emphasizing how the absence of controls in single‐arm studies amplifies uncontrolled confounders and underscores the need for future randomized trials to mitigate such heterogeneity.

The lack of heterogeneity in the meta‐analysis of severe ADRs to MTX in IGM of 8 single‐arm studies (*I* ^2^ = 0.0%, Q‐test *p* = 0.6883) might be explained by a combination of methodological similarities, identically practiced clinical activities, and intrinsic traits of MTX tolerability that led to homogeneously rare event reporting as demonstrated by the resultant pooled proportion of 0.5% (95% CI: 0.03%–9.37%) in the random effects model and regular use of adjunctive supportive therapy, including weekly folic acid tablets, was effective in overcoming adverse effects of MTX, which led to zero or almost zero severe adverse events in a variety of studies [14, 17, 21]. The strict exclusion criteria of high‐risk patients such as malignancies, chronic infections, or other granulomatous diseases were also consistently applied so that the population of the baseline patients was homogeneous and reduced the risk through uniformity [17, 20, 21].

The heterogeneity observed in MTX protocols across the included studies is a notable limitation, reflecting the lack of standardized treatment guidelines for IGM as repeatedly highlighted by Akbulut et al. [3–6]. Dosing varied significantly, ranging from 5 mg/week to 25 mg/week, administered orally or subcutaneously. In direct response to expert calls for MTX‐specific standardization in IGM [3, 4], while the existing literature does not provide a definitive consensus, our meta‐analysis offers some insights that could inform a potential standardized protocol. Based on the dosages and outcomes from the included studies, a weekly MTX dose of 7.5–15 mg is most commonly reported [13, 16, 18, 21]. This range appears to be a reasonable starting point. Our findings also suggest that combination therapy may be more effective than monotherapy for achieving complete remission. Therefore, we propose that an initial regimen of MTX at 7.5–15 mg/week in combination with a tapering course of corticosteroids could be considered a standard first‐line approach in patients with corticosteroid resistance or dependence. Furthermore, several studies noted a strategy of escalating the MTX dose or switching to subcutaneous administration in cases of nonresponse or intolerance [16, 17, 19]. It is recommended to approach the treatment having in mind a certain flexibility. For those patients who after a certain period show no or only partial response—say 3–6 months—are candidates for either increasing the dose to 15–25 mg/wk or converting to subcutaneous administration. Although our data cannot firmly define an ideal treatment duration, some reports suggest regimens lasting from 6 to 24 months with a median of just over 1 year [17, 19, 21]. We recommend a minimum treatment duration of 6–12 months in order to achieve and sustain remission with a gradual tapering of MTX dosing in conjunction with symptom resolution, followed by long‐term monitoring for recurrence. This proposed protocol directly addresses the gaps in MTX application noted in Akbulut’s reviews of > 3,000 GM cases [5] and aligns with their reported successful regimens in smaller IGM cohorts [3, 4]. This proposed protocol, grounded in the aggregated evidence from this meta‐analysis, provides a more structured framework for clinicians. Future randomized controlled trials with standardized protocols are needed to validate this approach.

This meta‐analysis provides valuable clinical context by synthesizing the current evidence on the efficacy and safety of MTX for IGM, the idiopathic subset of GM, a rare and complex condition with a significant clinical impact [22–26]. The findings reinforce the role of MTX as a viable therapeutic option, especially for patients with a difficult‐to‐manage disease course. While IGM is often challenging to diagnose and can mimic inflammatory breast carcinoma [27], our results, drawn from a synthesis of global studies, show that MTX achieves complete remission in 61.6% of patients (95% CI: 40.4%–79.2%), particularly when used in combination with corticosteroids. Crucially, the analysis on severe ADRs highlights a key clinical advantage: the low incidence of severe side effects, suggesting that MTX is a well‐tolerated treatment for IGM. This tolerability is essential for a chronic condition that may require long‐term management.

MTX has also become an effective treatment agent in IGM, especially when patients do not respond to corticosteroids or when immunomodulators are to be used long term. It works well because it involves a complex mechanism of action, which helps to address major pathways in the inflammatory cascade. MTX acts mostly as a folate antimetabolite, which blocks the dihydrofolate reductase and blocks the production of DNA and RNA, thus limiting the multiplication of inflammatory cells [28]. In addition to this, it modulates immunomodulatory properties that suppress T‐cell activation and suppress proinflammatory cytokines such as TNF‐a, IL‐1b, and IL‐6 [29]. MTX also augments the production of the anti‐inflammatory adenosine [30], angiogenesis inhibition by inhibiting VEGF [31], and inflammatory cell apoptosis [32]. It also has antifibrotic effects that could help curb the overgrowth of scar tissue in IGM [33]. Although the exact mechanism of action is not clearly understood, MTX can possibly suppress proliferation of fibroblasts as well as collagen, which can reduce fibrosis in IGM; nevertheless, the optimal dosage and the period of MTX use are yet to be clearly defined [34].

## 5. Limitations

This meta‐analysis has several significant limitations on the results, which could affect their interpretations. To start with, the number of studies included is small, especially in the meta‐analysis of recurrence, and complete remission, which comprised of four and eight studies, respectively. This is a very small sample size, and most studies were of a single‐arm nature that does not allow us to conclusively determine the effectiveness of MTX. Second, the research had a high level of clinical and methodological heterogeneity. As it was mentioned in the discussion, there was a broad range of dissimilarity in the selection of patients (e.g., newly diagnosed vs. corticosteroid‐resistant), treatment protocols (monotherapy vs. combination therapy, different doses, and durations), and definitions of the major outcomes (e.g., complete remission, recurrence). Such considerations have contributed significantly to the high statistical heterogeneity in the analyses of efficacy and render the pooled results less credible. Despite the conducted subgroup analyses, they were constrained in terms of the small size of the studies per subgroup and the high heterogeneity of the subgroups themselves did not allow making strong comparisons. Third, most of the studies included were retrospective; control groups did not exist and were single‐center and case series designs. This design will be prone to selection bias and confounding since the patients might have been selected to receive MTX therapy because of unmeasured factors that may affect the results.

## 6. Conclusions

The meta‐analysis offers an all‐inclusive review of the effectiveness and safety of MTX in the management of IGM. We find that MTX is an active agent in attaining complete remission, combination therapy has a greater complete response rate of 61.6 (95% CI: 40.4–79.2), and corticosteroids may be more effective in combination therapy. The tolerability of this treatment can be seen in the risk of the critical side effects that result in MTX discontinuation being low.

**Nomenclature**
IGM: Idiopathic granulomatous mastitis
MTX: Methotrexate
PRISMA: Preferred Reporting Items for Systematic Reviews and Meta‐analyses
PICO(S): Patient(s), intervention, comparator, outcome(s), study design
NOS: Newcastle–Ottawa Scale
AMSTAR: Assessing the Methodological Quality of Systematic Reviews
OR: Odds ratio
CI: Confidence interval
ADRs: Adverse drug reactions
MeSH: Medical subject headings

## Author Contributions

Ying Han: conceptualization, methodology, data curation, formal analysis, writing–original draft, writing–review and editing, supervision, LiHui Shi: data collection–review and editing, funding acquisition.

YanRan Zhang: data curation–review and editing.

## Funding

This work was funded by the Science and Technology Project of Beijing Tongzhou, KJ2024CX015.

## Conflicts of Interest

The authors declare no conflicts of interest.

## Data Availability

Data sharing is not applicable to this article as no datasets were generated or analyzed during the current study.
